# Children’s literature to promote students’ global development and wellbeing

**DOI:** 10.15171/hpp.2020.05

**Published:** 2020-01-28

**Authors:** Manuela Pulimeno, Prisco Piscitelli, Salvatore Colazzo

**Affiliations:** ^1^PhD Candidate in Human Relations Sciences, University of Bari “Aldo Moro”, Bari, Italy; ^2^UNESCO Chair on Health Education and Sustainable Development, Federico II University, Naples, Italy; ^3^Department of History, Society and Human Studies, University of Salento, Lecce, Italy

**Keywords:** Children, Literature, Storytelling, Fairytales, Health, Wellbeing, School

## Abstract

**Background:** Tales were transmitted from one generation to another, enriching young people with values, beliefs, imagination and creativity. Children’s literature still plays a crucial part in education as it provides knowledge and entertainment, representing a typical example of "edutainment". In this paper, we carried out a review to examine pedagogic, didactic and psychological/therapeutic dimensions of children’s literature, with the aim of highlighting its role in promoting students’ holistic development and wellbeing.

**Methods:** We have searched for original articles (from 1960s to 2019), by using the following keywords: "fairytales" or "fairy tales" or "folktales" or "fables" AND "education" or"development" or "learning" or "teaching" or "school" or "curriculum" or "classroom" AND "children" or "child" or "kids" or "childhood" AND "health" or "wellbeing".

**Results:** We found 17 studies concerning pedagogic aspect of children literature, while 21 and17 studies were selected for didactic and therapeutic dimensions, respectively. From a pedagogic point of view, tales convey basic values useful for children lives. In a didactic perspective, properly chosen storybooks represent a valuable resource for school activities, improving students’ language skills and building up a friendly/respectful classroom environment. Children stories are also used by health professionals for therapeutic purposes (bibliotherapy) to prevent unhealthy habits and addictions, or address psychosomatic disorders. Finally, storybooks and web-based/digital stories can be an effective vehicle for health contents, to encourage the adoption of healthy lifestyles among schoolchildren.

**Conclusion:** Children’s literature and storytelling could be helpful in promoting students’ global development and wellbeing, when included in school curricular activities.

## Introduction

Myths, fables and fairytales – originally founded on oral tradition – allowed adults to communicate with young people in an uninterrupted process until nowadays.^1^ Tales have been told everywhere and in every time to educate, entertain and increase individuals’ awareness about moral principles and customs, thus representing an important part of traditional heritage as well as a way to reinforce tolerance and mutual knowledge among different populations.^2^

Reading or listening to tales can be considered significant community practices, capable to impact on young generations, empowering and preparing them for the future.^3^ Since culture is crucial for learning, stories have a fundamental part in shaping individual’s role in the society, becoming a helpful resource from didactic, psychological/therapeutic and pedagogic perspectives.^4^

From a didactic point of view, storybooks can provide children with new information about the world, enrich vocabulary and enhance specific language skills (in the classroom or at home), nurturing communication between the storyteller (teacher, parent or other professional staff) and the listeners.^5,6^

It is known that stories – by reproducing fictional situations that match with children’s real problems – allow them to feel comfortable and safe in difficult circumstances, ensuring emotional security and providing healthier ways to deal with internal struggles, life adversities and stressors.^7^ Story-tales compensate what young people may lack, by presenting them positive patterns of behaviours and constructive models through the characters they could identify with.^8^

Storybooks (or digital tales) are easier to understand for all children compared to abstract notions or theories, and might become special instruments for mapping the reality and conveying health contents, especially to the most vulnerable groups.^9,10^

As suggested by the World Health Organization (WHO), health literacy should be incorporated in school curricula, in the context of a health-promoting classroom environment, in order to provide new generations with useful knowledge about healthy lifestyles.^11-13^ Actually, school represents the ideal setting to perform health-related interventions and positively influence students’ wellbeing as well as their academic achievements.^14-16^ The final goal is to involve young generations in practical actions about healthy habits (i.e. balanced nutrition and physical exercise) and prevention of risky behaviours (such as cigarette smoking, alcohol consumption, drug use) through a personal re-elaboration of health knowledge.In our previous systematic review, we have provided evidence for taking into account narrative-based strategies among the possible highly motivating approaches to encourage schoolchildren in adopting healthy eating habits since childhood.^17,18^ More broadly, in this paper we explored the rationale for using children’s literature and storytelling in school setting to promote students’ global development and wellbeing.

## Material and Methods

A narrative review has been carried out in order to analyze the pedagogic, didactic and psychological/therapeutic dimensions of children’s literature, highlighting the potential of narrative-based strategies in fostering students’ global development and wellbeing. Starting from January 2019, over a five-month period in the context of PhD in Human Relations Science of Bari University (Italy), we have searched on Web of Science for original articles and books, published from 1960s to 2019, by using the following keywords: “fairytales” or “fairy tales” or “folktales” or “fables” AND “education” or “development” or “learning” or “teaching” or “school” or “curriculum” or “classroom” AND “children” or “child” or “kids” or “childhood” AND “health” or “wellbeing”. We summarized definitions of health, presenting “wellbeing” (in its three dimensions of physical, emotional/mental and social health) as the main goal of every educational practice, and school system as the ideal setting to display health-related interventions. We also used citation tracking to detect other papers concerning children literature and narrative-based strategies (from oral storytelling to printed books and digital resources) as effective operational tool for conveying health contents to promote global development and wellbeing in school setting, along with the prevention of risky behaviours. Finally, we have provided brief definitions of children’s literature, presenting some historical insights about its pedagogic or didactic use, and psychological/therapeutic applications (bibliotherapy and narrative medicine).

## Results

Children’s literature is broadly defined as any creative literary work that has been especially written and designed for children’s use.^19^ Only in the 18th century, with the evolving of the concept of childhood, a separate genre of children’s literature was created.^20^ Modern children’s literature comprises short fairytales and fables, picture books, comics, cartoons, novels, nursery rhymes that can be potentially appreciated by most children.^21^ In our search, we selected 17 studies concerning pedagogic dimension of children literature,^20,22-37^ while 21 and 17 studies were chosen as addressing didactic^1,5,38-56^ and therapeutic dimensions,^6,7,57-71^ respectively (Table 1).

### Children’s literature as narrative tool in education: pedagogic dimension

The crisis we are facing is not only economic and financial, but also political, cultural and ethical, generating anxiety and fear due to the perception of a precarious existence in the context of a growing individualism and insensitivity to other people’s difficulties. Moreover, our society measures everything in terms of monetary value, giving priority to scientific/technological knowledge and decreasing the relevance of human sciences, which have nurtured the traditional humus of citizenship education.^72^

Despite educational system is dealing worldwide with several challenges, school still represents the ideal setting to display interventions aimed at promoting students’ holistic development. Beyond its specific commitment, it is essential to build up healthy, respectful and satisfied citizens: the future adults capable to take care about themselves, the others and the environment.^24,73^

In the globalization era, characterized by deep socio-economic changes and collapse of the traditional social tissue (i.e. new forms of poverty, increase of inequalities, family mobility etc.), the cultural heritage of folktales – easily available both for parental and teachers’ use – could represent a helpful tool for promoting individual personal growth, social cohesion and sustainable development.^2^

Tales were told and are still told in every society and in many different settings to share experiences, customs, norms, and values, providing the listeners with entertainment and new knowledge.^25^ In the “culturalistic” perspective, children’s stories belong to a specific cultural niche that could help young people to move into the life, allowing them to understand who they are as human beings and how they can contribute to the progress of the world.^26^

Children’s literature continues to be a significant opportunity of presenting moral principles in an enjoyable and engaging way^27^ and it is growing fast along with the aim to entertain, educate and provide new knowledge (in line with the new concept of “edutainment”), being able to integrate fun and adventure demanded by children (simulating the activity of free play) with the adults’ objective of offering them a set of moral examples.^20,28^

A big part of children’s literature is represented by fairytales, which have the final goal of transmitting the basic universal values, and raising children’s awareness on many aspects of the life.^29^ That’s why, even before printing press was invented, fairytales have been used by parents to transmit culturally appropriate moral norms to their children from an early age, equipping them with information, attitudes, and skills that could act as a kind of “vaccination” against all kind of threats to individual or collective health.^30^

The most famous example fulfilling these criteria can be found in “Pinocchio”, written by Carlo Lorenzini (Collodi) to make children aware about the consequences of adopting wrong behaviours.^31,32^ Similarly, in Germany, the Grimm Brothers presented noble values and positive models in their amazing adventures, helping children to understand what is good and what is bad.^33^

Tales are very interesting for children because they show real aspects of family and community life, reinforcing the relations with the parents and highlighting ethical values related to social life.^34,35^ Through implicit meanings embodied in the stories, children indirectly acquire pedagogical messages, able to influence their global personality and stimulate a social sense of duty.^27^

Children’s stories are the place of endless possibilities, so that young people can open their mind to wide horizons, generate new viewpoints, find possible alternatives or solutions to problems, cultivating their points of strengths such as self-confidence and resilience.^36^

The role and importance of children’s books have changed in modern society, but even today, children’s literature (including movies and digital resources) influences our daily lives and contributes to the development of young people in a number of ways, ranging from the transmission of values to didactic purposes. The presence of digital technology represents a challenge but also an opportunity for traditional fairytales’ or fables’ existence. Digital storytelling (the combination of the art of telling stories with a variety of multimedia tools) is a helpful instrument to generate more appealing and stimulating learning experiences.^37^

Actually, printed publications tend to be expensive, while the Internet-based resources are a cheap alternative (usually available online for free), and might raise children’s interest towards books in many different ways. Combining narrative possibilities and technological potentials can be more powerful in terms of access to information, sharing of work, differentiated and motivated learning models. However, there is a fundamental distinction (at least in terms of establishing good relationships with educators) between watching a fairy tale on monitors (static and passive approach or even by computer-based interactive mode) and listening to a live re-telling of it.^22,23,74^

### Didactic dimension of children’s literature

The didactic intention of narrative works was discovered on clay tablets in Sumerian and Babylonian texts, dated back many centuries before Aesop’s fables (successively put into Latin verses by Phaedrus). Myths initially transmitted orally became well-known throughout the Mediterranean area thanks to Greek manuscripts of Alexandrian scribes, who used them in their daily education activities. Also philosophers (i.e. Plato) introduced myths and fables in their academic lessons with students and disciples: the rules of grammar and style were learned through the stories, encouraging young scholars to create new ones. Fables of Aesop were considered as useful didactic means also in medieval schools to teach Latin and rhetoric.^1^

Even today, children’s literature – as integral part of primary school curriculum – could be a significant experience in the lives of children, with fables and fairytales being used as motivating teaching tools in both humanistic and scientific disciplines.^38-40^ Educators are aware that all creative and artistic activities, including literature – while entertaining listeners or readers – can play a fundamental role in improving students’ knowledge, but also in the acquisition of daily life skills, useful to cope with any problematic situations.^41^

Childhood is a crucial stage for language development,^75^ so it is important to make it a pleasing experience: reading or listening to stories could be a joyful way for language training, able to overcome all the possible learning barriers.^42-44^ Thanks to the recurring narrative passages intrinsic in the fairy tales’ or fables, child is able to deal with some complicated concepts or patterns, which require more repetitions to be better interiorized. That’s why tales are a valuable resource in teaching foreign languages and improving language skills (writing, reading, speaking and listening).^45^ The use of narrative in teaching foreign languages has been found to lower the level of anxiety, allowing students to take risks in the language classes, thanks to the familiarity with stories and the relaxing learning environment generated by storytelling. Therefore, telling or reading stories is a successful strategy to acquire grammar structures, syntax, new vocabulary, increasing oral/written competences, and therefore the ability to communicate effectively and successfully.^46^

By reading or listening to stories, students enhance their verbal proficiency and learn to accurately express their thoughts and feelings in everyday relations, making practice of peace-making skills (i.e. negotiation and discussion).^47^

Learning from stories can stimulate and offer promising insights in other areas of children’s cognitive development such as problem-solving and reasoning skills.^48^ Educators should awaken children’s interest towards reading and, at the same time, encourage them to use imagination, finding themselves inside the story; once children become attached to their favourite characters, they can reproduce them while playing, following the time chain and cause-effect relation of narrated events, so that the educational message of the stories can be better interiorized.^5,49^ Educators should also be aware about their own responsibility when selecting children’s books for didactic purposes (not necessarily following popular titles or “best sellers”), and read the stories in a caring and warm environment.^50^ Storybooks are accessible to students of all ages and can be borrowed from libraries or friends, while digital storytelling can be easily and quickly found on the Internet, even for free.^51^

Multicomponent narrative-based approaches integrate traditional tales or other specifically developed storybooks, with audio and video resources (including those available on the Internet), cartoons, animated films, puppets or scenic elements.^23,52,53^ Theatre reading or dramatization of children’s literature can be used at school to overcome the risk of short attention span of schoolchildren, and when dealing with difficult textbooks. Reader’s theatre in the classroom involve students as actors as they were really acting on the stage, while the teacher is guiding the scene and giving suggestions to the characters. In a study investigating the impact of readers’ theatre over six weeks, students assigned to the theatre class showed significant progress in reading level, compared to a control group who received more traditional literary and vocabulary education. The readers’ theatre class presented better fluidity in reading and expression, enriched vocabulary, and increased motivation compared to the control group.^54^ Finally, it can be said that storytelling activities (including reader’s theatre) in school setting represent innovative didactic experiences, capable to build up also health knowledge and promote students’ global wellbeing.^55,56^

### Therapeutic dimension of children’s literature

Children’s storybooks not only provide new knowledge – by enriching children’s vocabulary and enhancing their communication skills – but also ensure emotional support during problematic circumstances of the life. Encouraging children to overcome fears and inner conflicts, tales act as promoters for change, positively influencing their social behaviour.^57^

When parents or teachers provide children with a book, they usually hope that they will absorb the moral values that it contains.^58-60^ Actually, fairytales can produce positive effects on personality development, satisfying all psychological needs of the children such as contact, entertainment, and cognitive demand. In the Freudian perspective, assuming the absence of a well-defined superego and moral standards in childhood, fairytales are useful to show proper patterns of behaviours needed by children.^61^

Children’s literature – as a form of artistic creativity – presents a therapeutic potential for readers and listeners, in the same way that Greek tragedy was able to “heal” the spectators.^62,63^ In the vision of the cathartic role of literature, we can say that it may influence children through psychological mechanisms, primarily consisting in involvement, imitation, identification, insight and universalism. Story-tales could be used in school-setting for primary prevention programs with the ultimate goal of preventing risky behaviours among young people, thanks to the potential of creative and artistic means such as specifically-developed children’s storybooks. Actually, narrative-based approach as a teaching and learning strategy is omnipresent in the classrooms, but it is infrequently used to promote students’ health.^64^

Literature, as well as other forms of art (music, dance, drama, drawing, painting etc.) can be used to empower and motivate children towards the adoption of healthy behaviours, contributing to the improvement of pupils’ quality of life. The educational properties of the stories allow young people to accept their own differences, while showing how the characters of the tale cope with difficulties, enabling readers to enter in a fantastic world of entertainment, and – at the story’s end – to come back into reality in a comfortable way.^65^

The main goal of art therapy in education is the holistic human development, to be accomplished by working on imagination, curiosity, and creativity, which represent all the basic features to be preserved in children, along with the natural needs of joy and play.^66,67^ Artistic activities present also the potential of breaking down cultural barriers, actively involving the most vulnerable and marginalized children, as assessed in a study examining the effect of a creative expression program designed to prevent emotional and behavioural problems in immigrant and refugee students attending multi-ethnic schools.^76^ This vision has been already adopted by the famous violinist Menuhin and his Foundation, to help vulnerable and disadvantaged children by using music and other form of arts.^77^

Within the broad umbrella of art therapy, we can find “library therapy”, which S.M. Crothers in 1916 has turned into the term “bibliotherapy”, characterized by the fact that the treatment is carried out by the means of literature, using books to foster individual emotional wellbeing. Understanding the principles and practices of bibliotherapy is essential for teachers and educators, working with children, who may take benefit from the exposure to reading materials related to their specific problems.

The “healing” potential of books was known since the time of the ancient Greece and even before: Ramses II in Egypt identified a group of books in his collection as “remedies for the soul”. Aristotle and other Greek philosophers believed that literature could deeply heal people, while the ancient Romans recognized the existence of a relationship between medicine and reading, with Aulo Cornelius Celso explicitly associating the reading with medical treatment. This attitude towards therapeutic opportunities of books was cultivated even in the Middle Age and Humanism/Renaissance times, but also in the late eighteenth century books were proposed as a remedy for different types of illnesses. Today, literature is somehow considered as psychological therapy, especially in childhood, and even as a cure for psychosomatic disorders.^6^

In the therapeutic approach, bibliotherapy includes also discussion and reflection on the story’s topics that overlap with the individual needs and have an evocative function that relies on projection and identification mechanisms. Proper storybooks work as a strategy for attitudinal change and self-improvement, acting through a compensatory function in children who lack of positive experiences which are often missing in their family or community.^68^ Therapeutic reading can also represent a form of prevention as the readers acquire a more flexible mind to recognize problems and eventually ask for help. There are books that address questions concerning physical appearance, emotions and character traits, family relationships, and socioeconomic problems.^69^ Bibliotherapy can be also applied in the field of psychotherapy for the treatment of minor disorders, eating behaviours and some forms of addictions, from alcohol and tobacco to drugs and ludopathy.^7^

Narrative medicine, an emerging discipline in healthcare field – which embraces medicine, psychoanalysis and literature – is used to overcome individual traumatic experiences. It helps patients and health professionals to tell and listen to the complex and unique stories of illness through an active approach (subjects are invited to compose poetic or literary pieces) or passive mode (consisting in reading already existing pieces).^70,71^

### Efficacy of narrative-based strategies to promote health and wellbeing in school setting

Health is defined by WHO Constitution as “a state of complete physical, mental and social wellbeing”.^78^ WHO has demonstrated that many early deaths can be avoided if each stakeholder in the society takes its piece of responsibility in promoting healthy lifestyles.^79^ Health promotion and prevention represent two sides of the same coin being both focused on proactively maintaining people healthy.^80^ Primary prevention should start as early as possible and school has the opportunity to guide people since childhood on the right path towards healthy life. Actually, education and health are intertwined, and it is undoubtable that wellbeing has also a remarkable impact on students’ learning outcomes. School represents the ideal setting to convey proper contents about risk and protective factors^81^ by using motivating approaches (including “teaching narratively”), able to capture the interest of pupils and generate a harmonic and non-competitive learning environment.^82^ Narration can be regarded as an interesting way to trigger students’ motivation^82^ and develop a “narrative thinking”, which is fundamental for every human experience, including learning and interiorization processes.^83-86^ Specifically developed storybooks can foster children’s self-responsibility towards health and stimulate critical thinking about the consequences of adopting risky behaviours (i.e. unhealthy eating habits), thanks to psychological processes based on the identification with the characters of the stories.^17^ Actually, children literature and storytelling have been proved to be effective in specifically conveying health knowledge: the persuasive effects of narrative engagement have been illustrated in many researches and reviews.^87-95^ De Graaf et al have specifically performed a systematic review of 153 experimental studies on health-related narrative persuasion with a focus on the narrative characteristics as potential explanatory factors in the effectiveness to convey a health message.^87,88^ The results showed that stories that presented a healthy behaviour were more often associated with effects on the intention to adopt it, and stories with high emotional content were usually more effective, as well as the use of a first or second-person perspective in the text. No differences were observed between the media used for the narrative intervention (book or video etc.), while the familiarity of the setting and the way of displaying the health message in the narrative was found to be a promising persuasive factor.^88^ Shen and Han assessed 25 studies comparing narrative to non-narrative messages, showing a significant effect of narrative for primary prevention and detection of risky behaviours, but not for cessation of negative attitudes (e.g., quitting smoking).^89^ Zebregs et al included 15 studies that recorded positive persuasive effects of narrative.^91^ Braddock and Dillard metanalyzed 74 studies that compared narrative-based interventions to a control group that did not receive any relevant message.^92^ Their results showed that, compared to a baseline zero-effect, narrative had positive effects on story-consistent beliefs, attitudes and intentions. By reviewing 45 studies, Tukachinsky et al concluded that engagement with the narrative and its characters was positively related to attitudes and intentions implied by the narrative itself.^93^ Other authors have focused on the persuasive effects resulting from the “transportation” into a narrative world: when children read, they “enter” into tales and act out together with the characters.^94^ Dahlstrom et al have shown that it is important to consider whether the persuasive message is integrated in the causal structure of the narrative or not.^95^ Stories with two opponent main characters seem to have an impact on narrative persuasion in the context of social issues, while tales presenting a transition of the characters from unhealthy to healthy behaviour may be particularly beneficial.^90^ Moreover, the content and form of the narrative - such as characters, events, and the setting of the story - are very important: characters can be more or less similar to the readers, thus producing a different persuasive effect.^96,97^ A further dimension relevant to health-related “narrative persuasion” is the context of the presentation used in the narrative: an entertainment format where the reader is unaware that the narrative has a persuasive intention or a narrative frame in which the persuasive intent is more explicit.^98-100^ In addition to narrative characteristics, variables related to target recipients – like the predisposition to become engaged in narratives and prior knowledge of the readers – as well as the environment/situation in which the story is narrated may increase or reduce the engagement and effectiveness of narrative-based interventions.^101,102^ Most likely, the full process of persuasion is determined by the interaction of narrative, recipient and situational factors (such as noise in the environment) that can distract the student and decrease engagement. It should be emphasized that contents of the stories must be close to the children and the main character’s mental states needs to be as much as similar to the feelings of the child. Finally, it seems that multicomponent approaches including printed stories or tales told by a health educator in a face-to-face settings (i.e. live storytelling) can produce effects on beliefs, attitudes, intentions and even on the behaviours of recipients.^103^

## Discussion

Oral and written tales are part of a collective memory, maintained from one generation to the next as an intangible cultural heritage for the transmission of moral values (i.e. Homer’s epic poems). As emphasized by the UNESCO’s Convention for the Safeguarding of Intangible Cultural Heritage in 2003, folktales play a dynamic role in bringing people closer together, thus ensuring knowledge exchange among different cultures and increasing the respect for others in a tolerant peaceful way.

At the start of 21st century, school system faces new challenges worldwide, pushing educators to display innovative strategies in order to motivate students and engage them in stimulating and “transformative” learning.^104^ This perspective goes beyond the passive acquisition of knowledge, moving toward a more active, experiential and participatory approach to lifelong learning.^105^ The adoption of cooperative practices into daily classroom activities can contribute to the enhancement of students’ wellbeing, lowering the competition and anxiety due to the pressure of success, currently detectable among schoolchildren.^80^

To achieve these goals, narrative interventions may be considered as one of the possible strategies for teaching and learning because children’s stories create the comfortable atmosphere that is usually lacking in school setting.^5,106^

Since ancient times, myths, legends, fables and fairytales have supported individuals to understand who they are as human beings and the world around them, allowing people to map the reality through the use of words and language.^107,108^

From fairytales and fables – plenty of adventures, heroes, personified animals, enchanted forests and magical objects – children gain additional experiences, feelings and thoughts, learning to cope with inhibitions, vulnerability, and shyness. According to the psychoanalytical interpretation, children’s stories lead readers towards a deep level of consciousness, dealing with the fundamental human questions expressed in the language of symbols. Beyond its educational purposes, children’s literature can positively influence mental wellbeing, nurturing thoughts, feelings and behaviours of young generations.^63^

Stories – as a kind of creative form of art – help children to fight (like the heroes) for good things and success in their life, satisfying their spirit of play, spreading good mood, with benefits on physical health, mental brightness and moral virtue. These latter represent the three dimensions of wellbeing – pillars for the integral growth of the child – in the perspective of building up the future mature and socially active man.^109^

Children’s literature presents a strong pedagogical component and should be regarded as a real educational strategy with the potential of being incorporated into school curricula. Learning experiences carried out in a friendly school environment generate improvement of emotional health and better academic achievements^8,110-116^ A properly chosen book stimulates children’s power of observation, reason, memory and imagination, broadening the range of experiences, compelling the readers to reflect on their behaviours, and find out possible solutions to their troubles while providing entertainment. A famous sentence of Albert Einstein was: “If you want your children to be intelligent, tell them fairytales; if you want your children to be more intelligent, tell them more fairy tales”.^117^ Unfortunately, in today’s busy society, adults lack the time to talk with children, so that reading or telling stories could represent a great opportunity of constructive exchanges in the family and at school.^28^

Multicomponent narrative-based approaches (storytelling, role-playing, games, post-reading activities) are able to satisfy children emotional needs, provide sensory input, increase attention span, and shape the aesthetic taste.^43,44^ The power of listening and speaking is able to create artistic images and induce schoolchildren to produce their own stories or tales. This is well accomplished by introducing them to literature from early childhood, and ensuring them interesting, funny and attractive materials. Telling or reading a story is cheap, pleasing, inclusive, it can be used in any setting without special equipment except the imagination.^50,118^ Moreover, it generates catharsis, resulting in reduced anxiety, better comfort, self-esteem, thus helping young people to cope with any adversity and improving communication of feelings.

According to Richard-Amato,^119^ students find themselves in the characters or narration, and learn how to behave adequately while facing similar situations in the future life.^6^ It happens that the child becomes aware about the topic of the story, unconsciously solves the problems, increasing self-confidence, with positive implications for personality development.^119^ By developing the imagination and creativity, children can discover new ideas and increase personal motivation to achieve their objectives. Albert Einstein was used to say: “When I examine myself and my methods of thought, I come to the conclusion that the gift of fantasy has meant to me more than any talent for abstract or positive thinking”.^117^

Finally, it can be considered also the contribution of literature to stimulate individual agency, applying the already acquired knowledge, to make the world more fit to human needs.^120^ In this perspective, the Italian writer Leonardo Sciascia was used to say that if he did not believe that literature could produce a change, he wouldn’t have continued to write.^121^

Stories are also able to convey health information about prevention of communicable and non-communicable diseases. Several researches highlight the role of storytelling as a source of beneficial effects in primary prevention. Reading a storybook or listening to stories is helpful for children as it promotes pupils’ emotional expression and psychological wellbeing; it can be used to stimulate changes in young people lifestyles, encouraging them in practicing physical activity and reducing the consumption of sweets and soft-drinks, ultimately resulting in a measurable reduction of body mass index in specific cohorts.^122^

Bibliotherapy facilitates behaviour´s externalization, promotes empathy and prosocial behaviours, and helps solving problems such as bullying and teasing, which represent common situations in every school. Several studies have demonstrated the effectiveness of treating bullying through bibliotherapy, that can become an innovative approach to promote a respectful school environment. A huge amount of children’s stories has been used in order to prevent and give other perspectives on bullying, demonstrating that children’s books can serve as a useful channel of exchange between parents, teachers and children. An example for that is the Child Adolescent Teasing in Schools (CATS) book review project and website, where – after the exposure to a fictional story about teasing and bullying – children share their own experience and are guided to develop successful coping strategies against teasing and bullying occurring at school.^123^

Children’s books have been also used to let students learn peaceful alternatives to the violence of modern society, focusing on conflicts prevention in the classroom and the way for overcoming the problem.^124^ This has led to the creation of specific lists of books which help children to better understand and cope with some situations of discomfort such as traumatic stresses.

“Therapeutic libraries” have been established for paediatric patients or their families in hospital setting according to the vision that literature can help children to improve their quality of life, reducing stress and pain levels associated with the hospitalization process.^125^

In a randomized trial, a combination of storybooks and workshop sessions have been successfully tested in primary prevention programs for anxiety management, showing a significant improvement in coping skills and perceived self-efficacy: every session was based on a story describing characters facing common stressors and how they deal with their daily problems.^126^ It can be said also that children’s literature offers strategies to overcome the anxiety and the fear of the unknown, stimulating reflection and re-elaboration of personal criteria to be applied in real life.^127^ Bibliotherapy is used in school setting (from primary to high school) to foster social and emotional growth, offering the opportunity to find a deeper understanding of self, solutions to personal problems and enhanced self-image.^128,129^

Finally, as demonstrated by the worldwide success of self-help manuals, bibliotherapy could be a helpful resource to reduce unhealthy food habits for the prevention or treatment of obesity, as well as in supporting who want to quit smoking or other addictions both in young people and adults.^130-132^

Limitations of this work are mainly due to the initial design of the study, representing an exploratory work that found few on-field experiences concerning the use of narrative-based strategies to promote health and wellbeing among schoolchildren. A future work, carefully planned as systematic review, could take advantage from the findings of this first attempt, in order to better refine a comprehensive search in scientific literature.

## Conclusion

Children’s literature offers young people the possibility to acquire a system of values (educational role), to be engaged in motivating learning activities (didactic aspect), and to deal with inner conflicts and life difficulties (psychological value). Based on international evidence, children’s literature and specifically developed storybooks can encourage the adoption of healthy choices and represent a useful preventive tool to foster young people’s global wellbeing, helping them to better cope with emotional/social problems while proposing proper patterns of behaviours and conveying health contents.^133-136^ Children’s literature is a helpful tool to “educate”, “teach” and “heal”, so that narration could be considered among the possible educational strategies which can be used for pedagogic, didactic and therapeutic applications in the promotion of children’s global development both at home and at school.

## Implications for practice

This review indicates that children’s literature not only presents a strong pedagogical and didactic value, but it can also generate benefits for global development and wellbeing of young people. Moreover, children’s literature can be regarded as a flexible instrument that facilitates the transmission of health contents to the students, allowing teachers to become “health educators”. Narrative-based strategies have the potential to be integrated as useful approach in the school curricular activities to specifically convey health contents (at least in primary and secondary school).^135,136^ Being able to impact emotional experiences and individual motivation, children’s literature should be considered as a powerful educational tool also for health professionals, who can take advantage from the use of stories to spread health information. Actually, narration is a “transformative” mean that can be useful – in the frame of educational contexts – especially for the prevention of obesity, risky behaviours and addictions (cigarette smoking, alcohol, drugs, bet). Finally, beyond the possibility to prevent future diseases at individual level, thanks to well-designed narrative-based interventions in school setting, children can become themselves “health promoters” and “inter-generational multipliers” of desirable effects by influencing in a positive way their families and community. In this perspective, the use of children’s literature to convey health contents and promote wellbeing in school children might represent an interesting instrument to foster collective health.

## Ethical approval

This review did not need any formal approval from ethical committee.

## Competing interests

There are no competing interests concerning this article. This research has been carried out in the frame of institutional activities of the PhD in “Human Relations Sciences” of Bari University and UNESCO Chair on Health Education and Sustainable Development, without receiving any external funding or economical support from third parties.

## Authors’ contributions

MP, PP and SC have conceived, prepared, written, approved and revised the manuscript.

## Acknowledgments

Authors are grateful to Prof. G. Mininni, Director of PhD Course in Human Relation Sciences at University of Bari “Aldo Moro”.

**Table 1:** Main selected studies concerning pedagogic, didactic and therapeutic dimensions of children literature

**Pedagogic Dimension**	**Didactic Dimension**	**Therapeutic Dimension**
Lesnik-Oberstein, 1998^20^	Steadman & Palmer, 1997^1^	Bettelheim, 1991^57^
Ohler, 2006^22^	Moyer, 2000^38^	Cairney, 1984^58^
Hunt, 2000^23^	Banister & Ryan, 2001^39^	Storr, 1986^59^
Zeece PD, 2004^24^	Riecken & Miller, 1990^40^	Purves & Monson, 1984^60^
Zipes, 1996^25^	Batini & Giusti, 2008^41^	Freud & Strachey, 1964^61^
Boyd et al, 2011^26^	Williams, 2000^42^	Bernays, 1979^62^
Hunt, 2006^27^	Daniel, 2013^43^	Heath et al, 2005^63^
Winnicott, 1964^28^	Brice, 2004^44^	Wyatt, 2008^64^
Nikolajeva, 1995^29^	Brown, 2000^45^	Piotrow & De Fossard, 2003^65^
Zipes, 2013^30^	Isbell et al, 2004^46^	Albert, 2010^66^
Kilpatrick et al,1994^31^	Mokhtar et al, 2011^47^	Reynolds et al, 2000^67^
Guroian, 2002^32^	Forgan, 2002^48^	Lenkowsky, 1987^68^
Zipes, 2002^33^	Apol, 1998^49^	Hoagland, 1972^69^
Yenika-Agbaw, 1997^34^	Zabel, 1991^50^	Charon & Eric, 2017^70^
Zeece, 1997^35^	Ohler, 2013^51^	Rudnytsky & Charon, 2008^71^
Robin, 2008^36^	Chai et al, 2010^52^	Babarro Vélez & Lacalle Prieto, 2018^6^
Seligman, 2009^37^	Unsworth, 2005^53^	Rozalski et al, 2010^7^
	Keehn et al, 2008^54^	
	Mallan, 1992^55^	
	Chard, 2000^56^	
	Johnson & Louis, 1987^5^	
